# Preferentially oriented BaTiO_3_ thin films deposited on silicon with thin intermediate buffer layers

**DOI:** 10.1186/1556-276X-8-62

**Published:** 2013-02-07

**Authors:** John P George, Jeroen Beeckman, Wouter Woestenborghs, Philippe F Smet, Wim Bogaerts, Kristiaan Neyts

**Affiliations:** 1 Department of Electronics and Information Systems, Ghent University, Sint-Pietersnieuwstraat 41, Gent, 9000, Belgium; 2 Department of Information Technology, Photonics Research Group, Ghent University, Sint-Pietersnieuwstraat 41, Gent, 9000, Belgium; 3 Department of Solid State Sciences, Ghent University, Krijgslaan 281, Gent, 9000, Belgium; 4 Center for Nano-and Biophotonics (NB-Photonics), Ghent University, Gent, 9000, Belgium

**Keywords:** Barium titanate, Buffer layer, Chemical solution deposition, Ferroelectric thin films

## Abstract

Barium titanate (BaTiO_3_) thin films are prepared by conventional 2-methoxy ethanol-based chemical solution deposition. We report highly *c*-axis-oriented BaTiO_3_ thin films grown on silicon substrates, coated with a lanthanum oxynitrate buffer layer of 8.9 nm. The influence of the intermediate buffer layer on the crystallization of BaTiO_3_ film is investigated. The annealing temperature and buffer layer sintering conditions are optimized to obtain good crystal growth. X-ray diffraction measurements show the growth of highly oriented BaTiO_3_ thin films having a single perovskite phase with tetragonal geometry. The scanning electron microscopy and atomic force microscopy studies indicate the presence of smooth, crack-free, uniform layers, with densely packed crystal grains on the silicon surface. A BaTiO_3_ film of 150-nm thickness, deposited on a buffer layer of 7.2 nm, shows a dielectric constant of 270, remnant polarization (2P_r_) of 5 μC/cm^2^, and coercive field (*E*_c_) of 60 kV/cm.

## Background

Barium titanate (BaTiO_3_ or BTO) thin films have been extensively studied over the years because of the wide range of applications in thin-film capacitors [1], non-volatile memories, electro-optical devices [2], and MEMS devices [3], owing to their interesting dielectric [4], ferroelectric [5], piezoelectric and electro-optical [6] properties. A variety of methods have been demonstrated for the growth of BTO thin films. Chemical solution deposition has gained wide acceptance because of its low capital investment, simplicity in processing, and easy composition control [7].

The epitaxial deposition of thin films on silicon substrates is a key technology for the development of small photonic and electronic devices, based on the current CMOS fabrication platform. The leakage current and optical scattering are expected to be much smaller for epitaxial thin films compared to polycrystalline thin films. However, the epitaxial growth of ferroelectric thin films on silicon substrates still remains a challenge. It has been reported that the deposition at elevated temperatures causes severe reactions at the thin film/silicon interfaces, resulting in silicate formation and degradation of the quality of the thin films [8]. Interdiffusion of silicon and the constituent elements at high temperature results in intermediate pyrochlore and secondary-phase formation rather than a pure perovskite phase [9]. Different methods have been proposed to use either a seed or buffer layer to promote crystal growth. Single-crystalline substrates as well as oriented thin films of MgO (1 0 0) [10], SrTiO_3_ (1 0 0) [11], LaAlO_3_[12], SRO/CeO_2_/YSZ [9], LaNiO_3_ (1 0 0) [13], and Pt/Ti/ SiO_2_[14] have been used to promote the growth of perovskite BaTiO_3_ thin films. Since the structure and orientation of the buffer layer can influence the subsequent ferroelectric thin-film growth, the deposition conditions and processing parameters play an important role [15,16].

In the present work, we demonstrate the growth of BaTiO_3_ thin films on silicon substrates by chemical solution deposition. A lanthanum oxynitrate buffer layer of 6- to 10-nm thickness is used as an intermediate buffer layer to promote the BaTiO_3_ crystal growth. We have studied the influence of the thickness and the heat treatment of the buffer layers and the deposition conditions of the BaTiO_3_ on the crystallinity, orientation, and morphology of the BaTiO_3_ films.

## Methods

### Buffer layer deposition

Polyvinyl pyrrolidone (45% in water) dissolved in 2-propanol is spin-coated onto the silicon substrate as an adhesion layer prior to the buffer layer deposition. Buffer layer solutions are prepared by dissolving lanthanum nitrate hydrate in 2-propanol. The solution is spin-coated on the silicon wafers at 3,000 rpm for 45 s and subjected to a heat treatment at 450°C for 5 min. Lanthanum nitrate hydrate (La(NO_3_)_3_) decomposes through nine endothermic weight loss processes with increasing temperature [17]. Between 440°C and 570°C, the lanthanum nitrate hydrate is decomposed to the intermediate-phase lanthanum oxynitrate (LaONO3). The thickness of the obtained buffer layers in this work ranges between 6 and 10nm as measured with ellipsometry.

### BaTiO_3_ thin-film deposition

Reagent grade barium acetate Ba(CH_3_COO)_2_ and titanium butoxide Ti(C_4_H_9_O)_4_ are used as precursor materials for barium and titanium, and glacial acetic acid and 2-methoxy ethanol are used as the solvents. The molarity of the solution is 0.25 M. The BTO precursor sol is spin-coated at 3,500 rpm for 45 s, followed by pyrolysis on a hot stage at 350°C to burn out the organic components. This leads to a film thickness of about 30 nm. This process is repeated three or four times to obtain a film thickness around 100 nm. Then, the silicon substrate with the BTO amorphous film is subjected to a high-temperature annealing at 600°C to 750°C for 20 min, with a tube annealing furnace in ambient air. The ramping rates for heating and cooling of the specimen in the annealing system are 100°C/min and −50°C/min, respectively. The process cycle (two or three spin coatings and subsequent high-temperature treatment) is repeated several times to obtain an oriented thin film with a thickness of a few 100 nm.

### X-ray diffraction measurements

The samples are first cleaned with acetone, isopropanol, and de-ionized water. The measurements are carried out with a D8 Discover diffractometer (Bruker Technologies Ltd., Billerica, MA, USA) with CuKα radiation. The diffractograms are recorded for 2*θ* angles between 15° and 64°, with a step size of 0.004° and time step of 1.2 s.

### Focused ion beam etching/scanning electron microscopy

The cross-section images of the specimens are prepared by a FEI Nova 600 Nanolab dual-beam focused ion beam system (FIB; FEI Co., Hillsboro, OR, USA) and an associated scanning electron microscope (SEM). It allows simultaneous milling and imaging of the specimens. The SEM column is equipped with a high-performance field-emission gun electron source, whereas the FIB system has a gallium liquid metal ion source.

### Atomic force microscopy

The surface roughness of the BTO thin films are measured by atomic force microscopy (AFM) analysis. The measurements are carried out with Molecular Imaging's Pico Plus modular scanning probe microscope system (Molecular Imaging Inc., Arizona, USA), in contact mode.

### *C*-*V* characteristics

Prior to the measurements, a top electrode is deposited with either chromium (Cr) or indium tin oxide (ITO; area 3.14 mm^2^, thickness 50 to 100 nm) by RF magnetron sputtering. A thin layer (15 to 30 nm) of ITO is used for the bottom electrode. The capacitance versus voltage (*C*-*V*) characteristics are measured with a HP4192 ALF impedance analyzer (Agilent Technologies, Santa Clara, CA, USA). The capacitance is measured for a small alternating current (AC) voltage which is superposed on a direct current (DC) voltage offset.

### *P*-*E* hysteresis measurements

A Sawyer-Tower circuit is used to measure the hysteresis loop in the polarization-electric field (*P*-*E*) diagram of the BTO films. The measurements are carried out at frequencies in the range of 100 Hz to 1 kHz with a sinusoidal AC voltage with an amplitude of 10 V peak-to-peak.

## Results and discussion

### X-ray diffraction analysis

**Figure 1:**
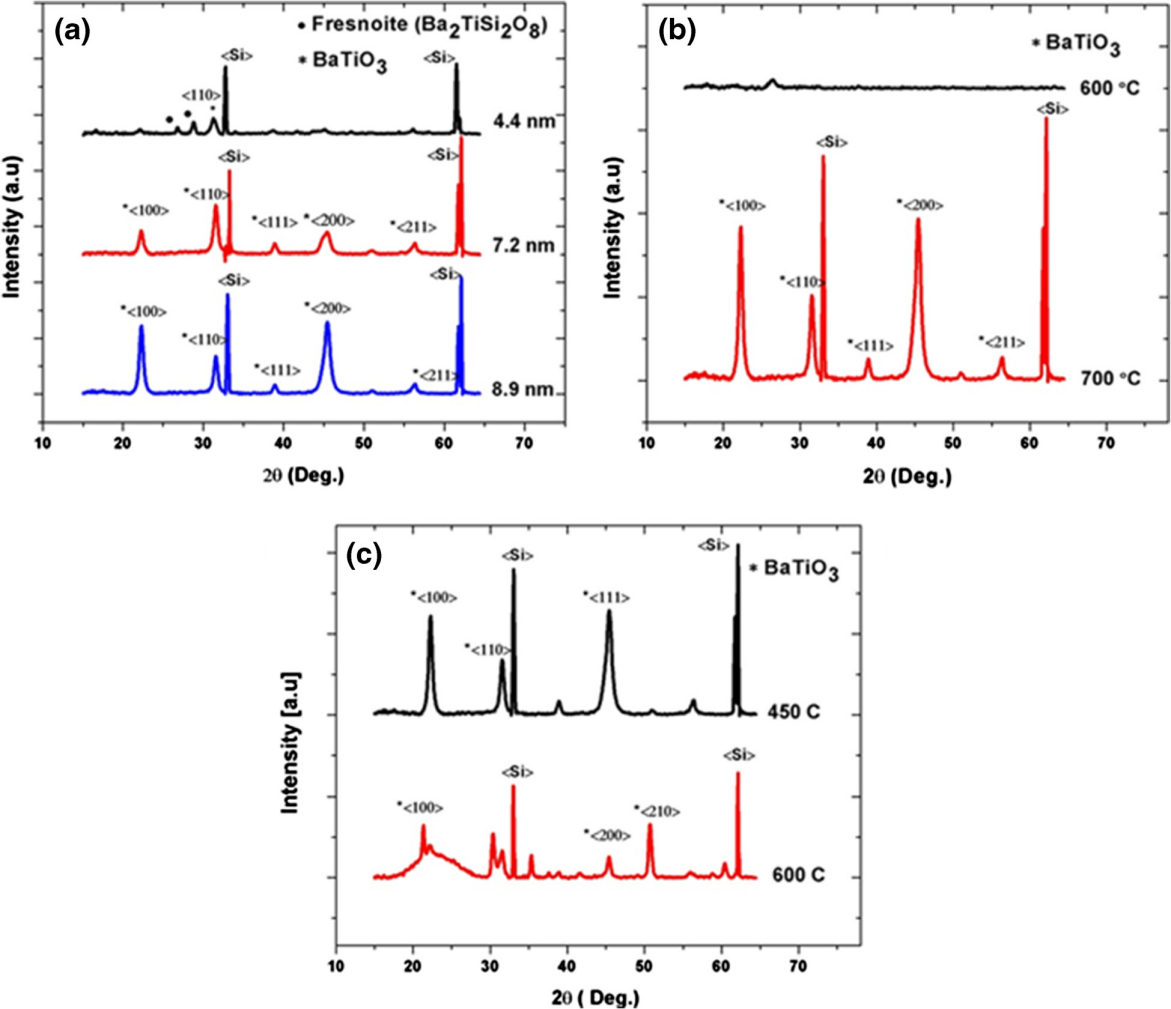
**XRD patterns obtained for the BTO thin films.** (**a**) BTO annealed at 700°C, with buffer layers of different thickness. (**b**) BTO annealed at different temperatures, with a 8.9-nm buffer layer. (**c**) BTO annealed at 700°C, with a 8.9-nm buffer layer, heat treated at 450°C and 600°C.

Figure 1 shows different X-ray diffractograms of BaTiO_3_ thin films deposited on bare silicon substrates and subjected to an annealing treatment at 600°C or 700°C. The thicknesses of the BTO films are determined as 150 ± 3 nm from spectroscopic (wavelength range approximately 300 to 1,500 nm) ellipsometry measurements. To analyze the films, we have used a multilayer system, where the buffer layer and BTO film (extraordinary and ordinary optical constants) are modeled with corresponding cauchy parameters. It is evident from Figure 1 that a minimum thickness of the buffer layer is necessary to prevent silicate formation at the Si-BTO interface and to promote crystal growth with a desired orientation.

Figure 1a represents a comparison between the BTO thin films deposited on silicon (annealed at 700°C) with different thicknesses of the intermediate buffer layer. When the buffer layer thickness is 4.4 nm, the secondary fresnoite phases (Ba_2_TiSi_2_O_8_) are dominant and only few diffraction peaks correspond to crystalline BTO. However, it is found from our experiments that a slightly thicker buffer layer of 7 nm is sufficient to yield well-defined diffraction peaks corresponding to stoichiometric BTO (BaTiO_3_), with a mixed <100> and <111> orientation. Even though a clear peak split is not observed at 45°, the broadened diffraction peak shows the possibility of a <002> BTO orientation. Any further increase in the buffer layer thickness leads to a stronger diffraction intensity along the <100> orientation. The increase in the buffer layer thickness reduces the strain energy within the BTO film and influences orientation of the film with a better <100> texture. However, the deposition of thicker buffer layer is limited because of the poor adhesion of the lanthanum nitrate buffer layer with the underlying PVP organic film. The X-ray diffraction (XRD) measurements indicate that the films are crystallized into a pure perovskite phase, with a tetragonal geometry.

It is evident from Figure 1b that no diffraction peaks are observed for the samples (buffer layer thickness 8.9 nm) annealed at 600°C, whereas it shows well-defined peaks for films annealed at 700°C. The films annealed at 600°C do not show any diffraction peaks of fresnoite or BTO, indicating the amorphous nature of the film. The peak observed around 26° correspond to La_2_O_3_. The absence of the fresnoite silicate phases also indicates that no reaction happened at the BTO/buffer layer interface due to the interdiffusion of Si. Figure 1c shows the XRD patterns of BTO thin films (annealed at 700°C) deposited on 8.9-nm-thick buffer layers that are heat-treated at 450°C or 600°C. It is obvious from the measurements that crystallization of the BTO films is influenced by the heat treatment of the buffer layer. Since the LaO(NO_3_) intermediate phase is only present up to 570°C, after which an non-stoichiometric unstable La(O)_1.5_(NO_3_)_0.5_ phase appears, it is clear that the LaO(NO_3_) phase exhibits superior properties as an intermediate layer. The heat treatment influences the nucleation mechanism of the BTO film and results in different diffraction peaks in the XRD spectrum.

#### Crystal orientation of BTO thin film

**Figure 2:**
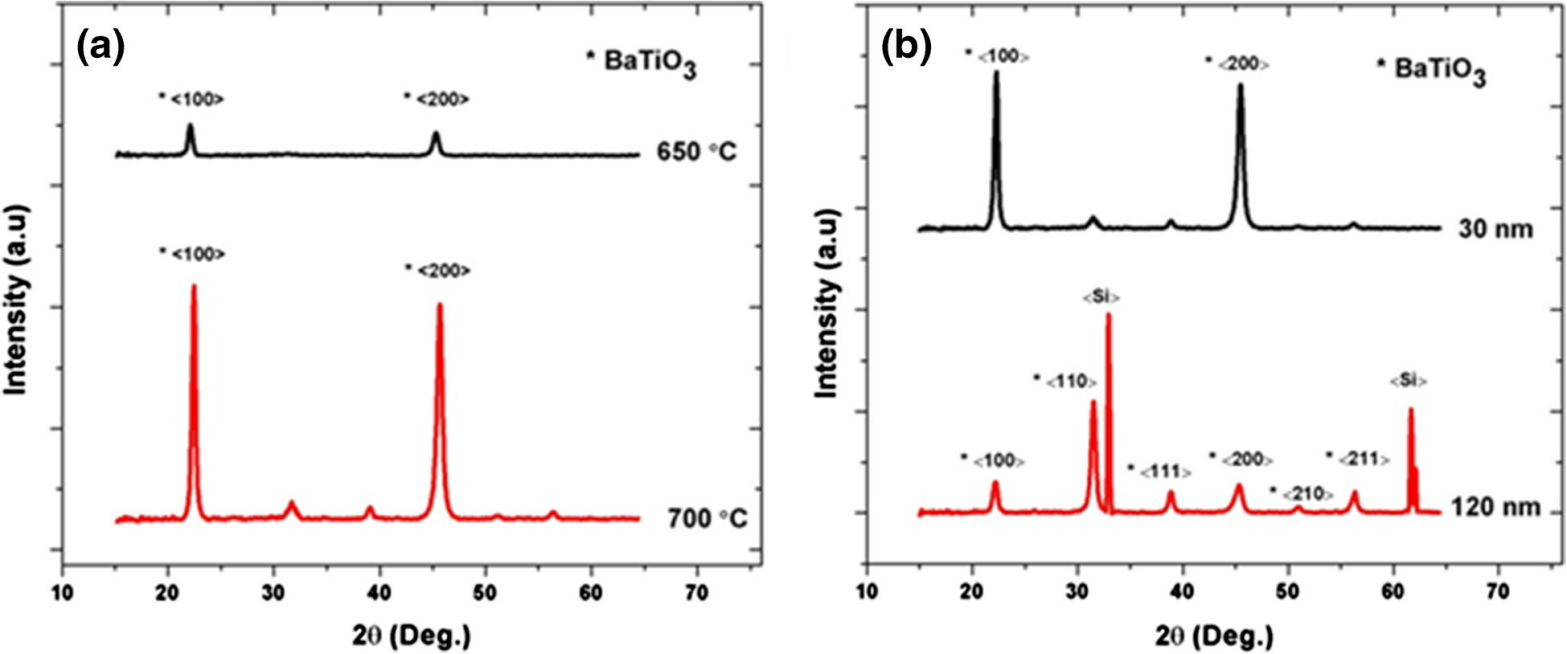
**XRD patterns obtained for BTO thin films.** The films were deposited on a buffer layer with a thickness of 8.9 nm and a BTO seed layer of 30 nm (**a**) annealing after each 30-nm BTO layer deposition at different temperatures and (**b**) annealing at 700°C after each 30-nm BTO layer deposition or after four 30-nm BTO depositions (120 nm).

The dielectric, piezoelectric, and electro-optical properties of the thin films depend strongly on the crystal orientation. Highly *c*-axis-oriented BTO thin films reported before are grown on either a single-crystalline oxide substrate or with a preferentially oriented thick (>100 nm) conductive or dielectric intermediate buffer layer [13,15]. The use of a thick buffer layer limits the performance of the ferroelectric films for certain applications (e.g., electro-optical devices). The results shown in Figure 2 indicate that we can grow highly *c*-axis textured BTO films with LaO(NO_3_) buffer layers (keeping the buffer layer thickness as 8.9 nm) by adding the number of annealing steps.

Figure 2 shows the XRD pattern of BTO films grown on a BTO seed layer. The seed layer is prepared by depositing a thin layer (30 nm) of BTO film on the buffer layer (8.9 nm), followed by pyrolysis (350°C) and annealing (700°C). After the seed layer, either the normal procedure is followed (annealing after 120 nm of BTO is deposited) or layer-by-layer annealing is used (after each 30-nm deposition). It is clear from Figure 2 that the BTO film grown over a BTO seeding layer has different crystallization properties, compared to the results mentioned in Figure 1. The BTO thin films grown with layer-by-layer annealing method show a preferential <100> orientation. The films annealed at both 650°C and 700°C show strong diffraction peaks along the <100> and <200> directions, with no sign of the secondary-phase silicate formation. It is evident from Figure 2b that the BTO films that are annealed after deposition of 120 nm of BTO (prepared by two to three spin coating and pyrolysis steps) show a stronger diffraction peak along the <110> direction (compared to the <100> direction). A comparison of the lattice parameters of the BTO film deposited on different buffer layers with bulk BTO crystal is mentioned in Table 1.

**Table 1:** Comparison of the BTO thin films deposited on different buffer layers with the bulk material

**Phase**	**Source**	**Method**	***a *= *b *(Å)**	***c *(Å)**	***c*/*a *ratio**
Tetragonal (p4mm)	Our work	Sol–gel	3.994	4.038	1.011
Tetragonal	On MgO buffer layer [18]	MOCVD	3.990	4.04	1.012
Tetragonal	BTO ceramic [19]	Chemical processing	3.998	4.022	1.0058
Tetragonal	BTO single crystal [20]	Chemical processing	3.992	4.036	1.011

### Microstructure and roughness measurements

**Figure 3:**
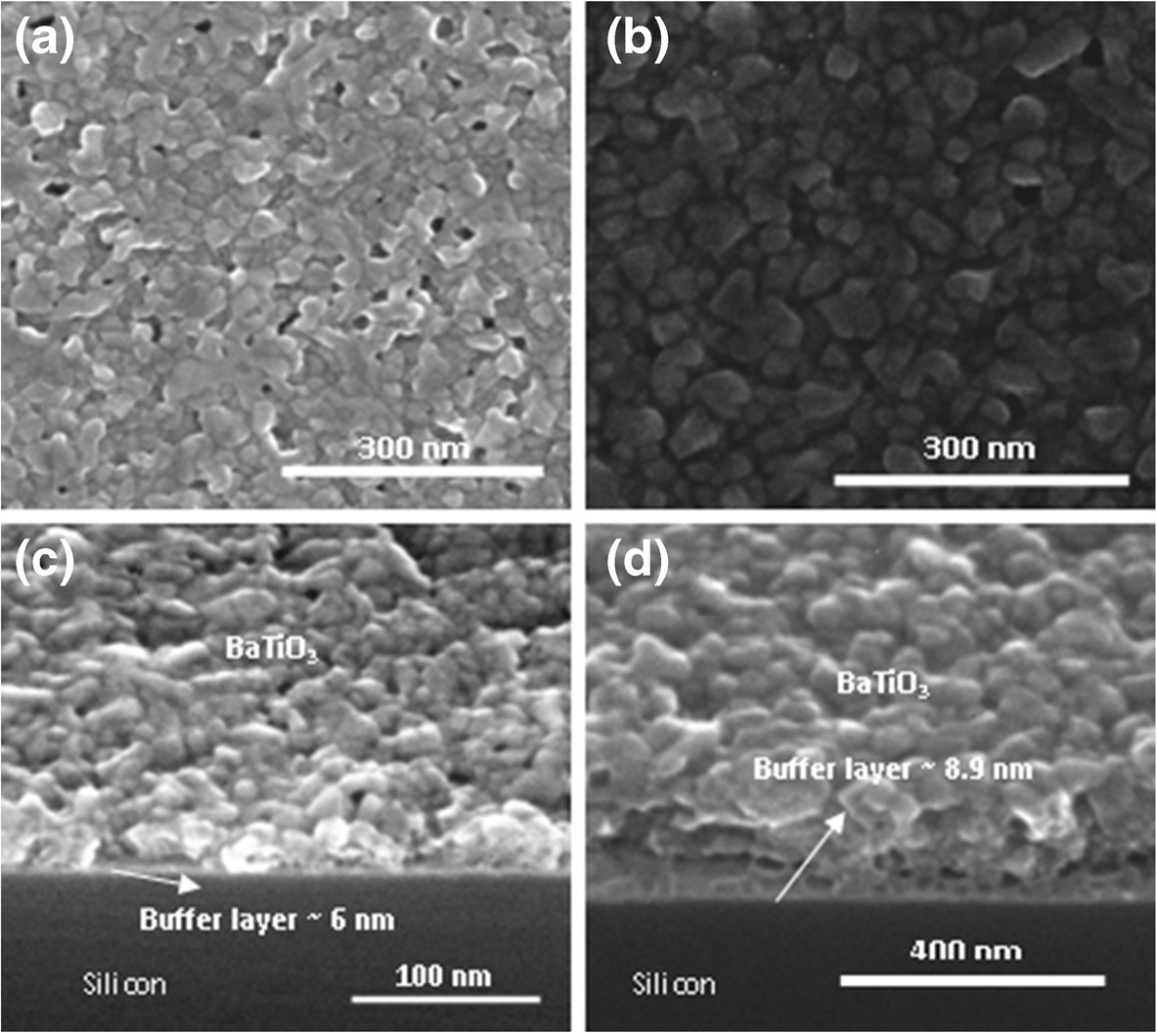
**SEM top view and cross-section images of BTO thin films.** SEM top view of BTO films annealed at 700°C, with buffer layers of (**a**) 6 nm and (**b**) 7.2 nm. Cross-section images of the BTO film deposited at 700°C (**c**) deposited with a buffer layer of 6 nm as shown in (a) and (**d**) prepared with layer-by-layer annealing for each 30-nm layer, with a buffer layer of 8.9 nm.

The SEM images of BTO thin films grown on silicon <100> substrates with different thicknesses of the lanthanum oxynitrate buffer layer are presented in Figure 3. The films annealed at 600°C (not shown) with buffer layers of different thickness are amorphous, and no distinct crystal grains are visible from the SEM measurements.

Figure 3a,b shows the top surface view of BTO films annealed at 700°C, with buffer layers of thickness 6 and 7.2 nm, respectively. The presence of the well-defined polygonal crystal grains is visible, and it shows the complete transformation of the amorphous films into a perovskite phase. The presence of the intercrystal voids in the BTO films (approximately 150 nm) deposited with buffer layers less than 6 nm is visible in Figure 3a,c. This increases the chance of electrical short circuit between the bottom ITO and the top evaporated Cr contact as we also experienced in the electrical measurements. However, the present work shows that the density of the intercrystal voids can be decreased to a great extent by increasing the thickness of the buffer layer to 7.2 nm. The films deposited with BTO seeding layers have further improved quality and appear to have a dense structure without the presence of pin holes (Figure 3d). It is also found that as the thickness of the BTO film before annealing is increased above 150 nm, the annealing process results in nano-cracks on the film surface.

The SEM cross-section images as shown in Figure 3c,d are prepared by cleaving the silicon sample. The cleaving causes rough edges, and the brittle nature of the thin film results in numerous regions without material. However, the presence of the thin buffer layer is evident, and the thickness matches with the data from ellipsometry measurements. The grain sizes of the films deposited at 700°C with a buffer layer of thickness of 7.2 nm are found to be between 30 and 50 nm, which is comparable to the other reported values [21].

**Figure 4:**
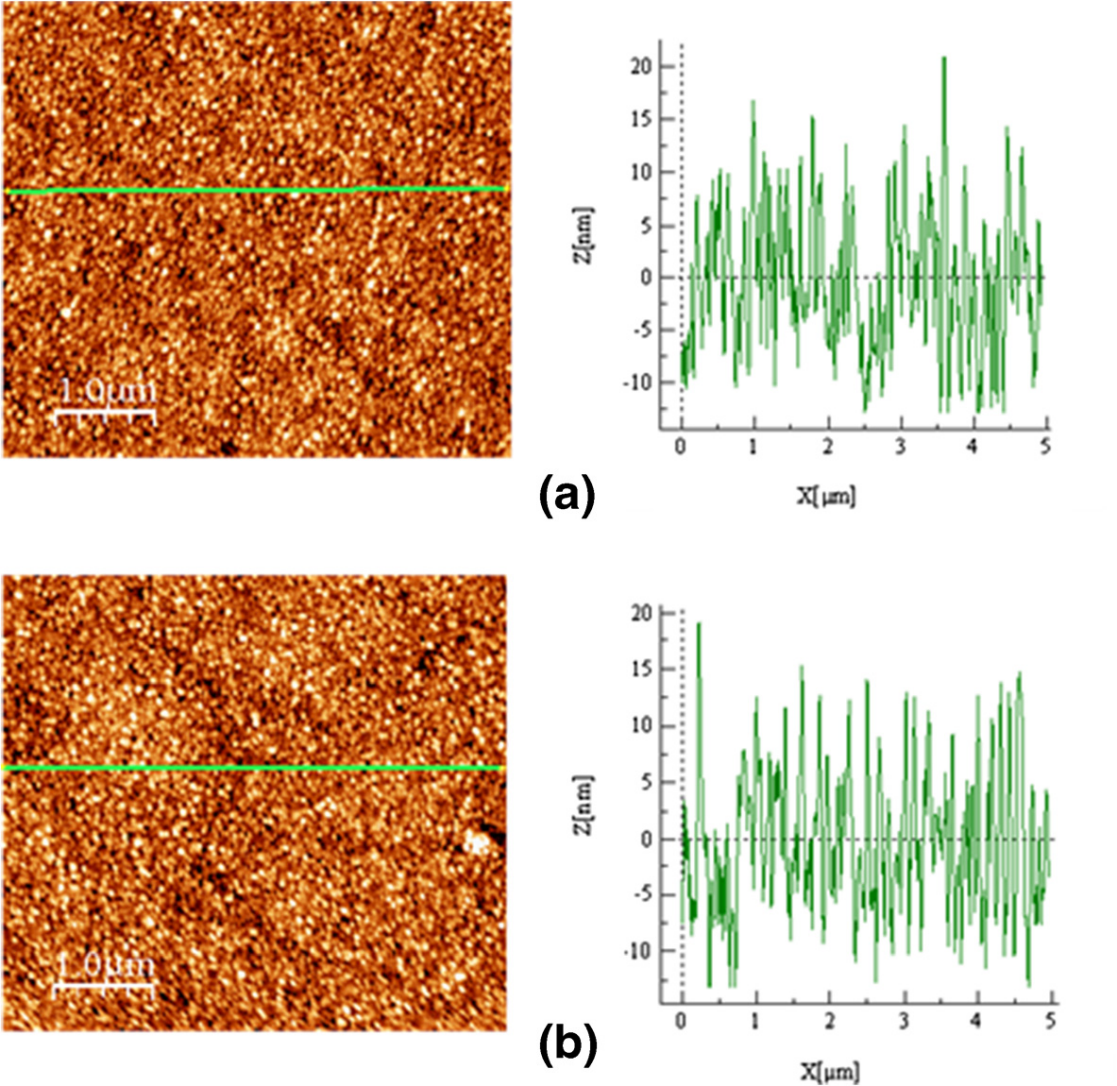
**AFM images of BTO thin films deposited at 700°C for different thicknesses of intermediate buffer layers.** (**a**) 6 nm and (**b**) 7.2 nm.

AFM measurements are carried out to estimate the roughness properties of the BTO films. The AFM images of the 150-nm-thick BTO films deposited at 700°C for different thicknesses of the buffer layers are shown in Figure 4a,b. The film deposited with the 4.4-nm buffer layer shows a roughness less than 10 nm, whereas the films deposited with buffer layers greater than 6 nm, show a larger roughness (10 to 15 nm) because of larger grain sizes.

### Dielectric and ferroelectric properties

**Figure 5:**
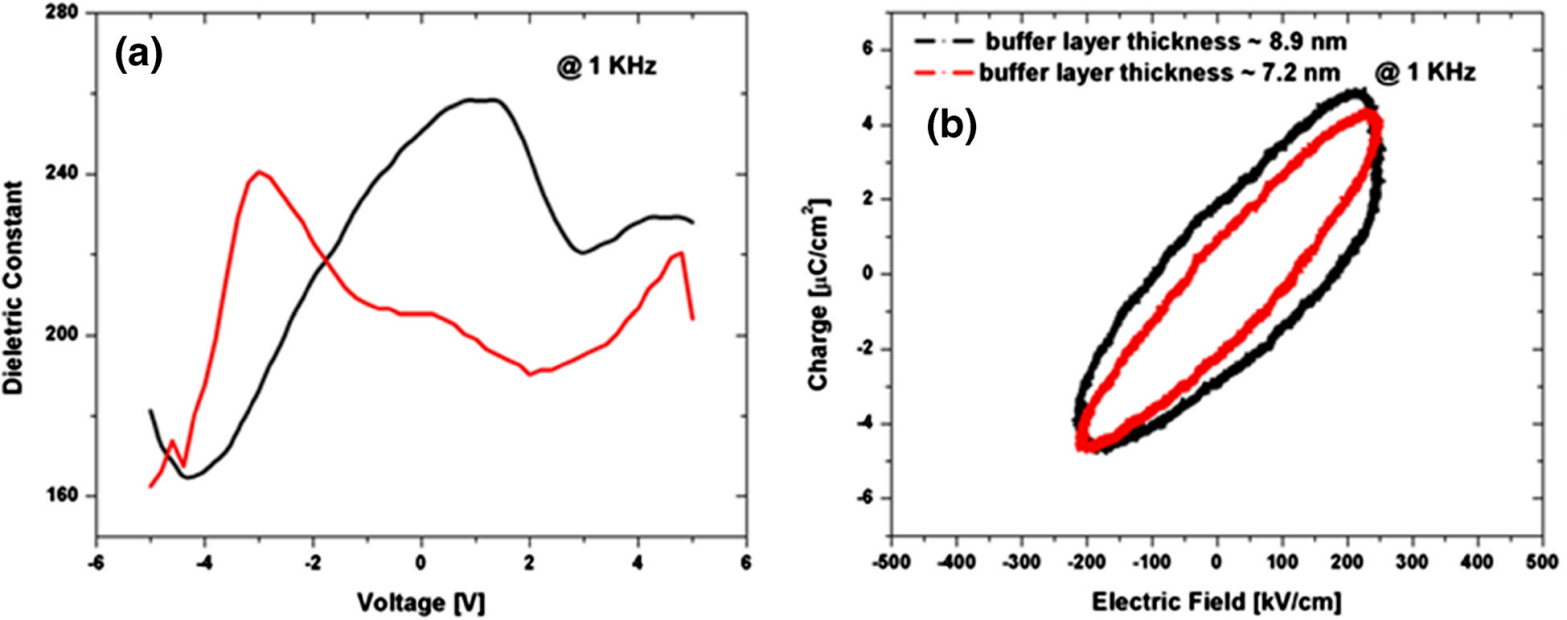
**AC dielectric constant and *P*-*E *hysteresis loop.** (**a**) AC dielectric constant as a function of the DC bias voltage for a BTO thin film (150 nm) annealed at 700°C with a 7.2-nm-thick buffer layer. (**b**) *P*-*E* hysteresis loop measured at 1 KHz with an AC voltage swing of 10 V-PP for the BTO films annealed at 700°C with buffer layers of different thickness.

The dielectric and ferroelectric properties of BTO thin films (thickness 150 nm, annealing temperature 700°C) grown on lanthanum oxynitrate buffer layers (thickness 7.2 nm or 8.9 nm, heat treatment 450°C) are estimated with *C*-*V* and *P*-*E* measurements. The *C*-*V* measurement shows the small signal capacitance as a function of a bias DC voltage (see Figure 5a). The butterfly shape indicates the ferroelectric hysteresis nature of the BTO tetragonal films. Two maxima for the dielectric constants are observed depending on an increase or decrease in the bias electric field.

The samples deposited with buffer layers below 6 nm often show electrical short circuit between the top and bottom contacts due to the intercrystal void formation. However, the highly oriented BTO films (150 nm) deposited on a BTO seed layer with buffer layers thicker than 7 nm, followed by layer-by-layer coating and annealing procedure (30 nm each time), show well-defined hysteresis loops. The BTO thin films (150 nm) appear to be stable, without breakdown up to electric fields of 400 kV/cm. The polarization of the films does not reach saturation due to the electrical breakdown at higher voltages. The films deposited with a 7-nm buffer layer show a dielectric constant of 270, remnant polarization of (2*P*_r_) 3 μC/cm^2^, and coercive field (*E*_c_) of 60 kV/cm, whereas the BTO film deposited on an 8.9-nm buffer layer shows a 2*P*_r_ of 5 μC/cm^2^ and *E*_c_ of 100 kV/cm.

## Conclusions

Well-crystallized BTO thin films are deposited on silicon substrates, coated with a 6- to 9-nm-thick lanthanum oxynitrate buffer layer. We have demonstrated that the thickness of the buffer layer is important for the crystallization, microstructure, and electrical properties of the subsequently deposited BTO thin film. We have also presented a method to control the orientations of the BTO films either by controlling the thickness of the buffer layers or by modifying the deposition procedure. A buffer layer of 6 nm is found efficient to prevent secondary-phase formation and to allow high-temperature deposition. The problems associated with the formation of the intercrystal voids have been improved by controlling the process as well as buffer layer parameters. The BTO films deposited on the 7.2-nm-thick lanthanum nitrate buffer layer show a relative dielectric constant of 270, a remnant polarization (2*P*_r_) of 5 μC/cm^2^, and a coercive field (*E*_c_) of 100 kV/cm, which make it a suitable candidate for future electronic and photonic devices. Although the electrical properties are not as good as reported elsewhere, we believe this is the thinnest buffer layer reported up to now which results in preferentially oriented and well-crystallized BTO thin films.

## Competing interests

The authors declare that they have no competing interests.

## Authors’ contributions

JPG performed the experiments and drafted the manuscript. WW designed the electrical measurement setup, and PFS carried out the X-ray diffraction measurements. JB and WB helped analyze the data and participated in revising the manuscript. KN supervised the work and finalized the manuscript. All authors read and approved the final manuscript.
